# Supplementary material for: A ligand-specific blockade of the integrin Mac-1 selectively targets pathologic inflammation while maintaining protective host-defense
Source: Nat Commun. 2018 Feb 6;9:525. doi: 10.1038/s41467-018-02896-8 (PMC5802769; doi:10.1038/s41467-018-02896-8)
Supplement: Supplementary file 1 — Supplementary Information [file 41467_2018_2896_MOESM1_ESM.pdf]

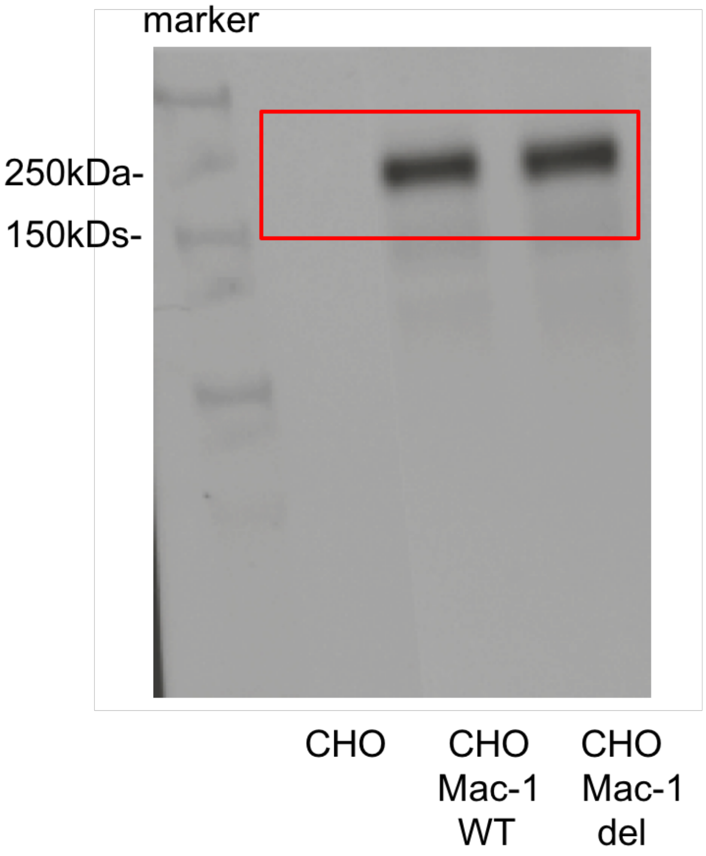

Supplementary Figure 1: Uncropped western blot membrane of Fig. 1B. Anti-M7 antibodies (10μg/ml) were incubated with lysates from native Chinese hamster ovarien (CHO) cells, CHO cells over-expressing native human (Mac-1 WT) and permanently activated human Mac-1 (Mac-1 del) in a Western Blot. CHO cells were generated as described before[1].

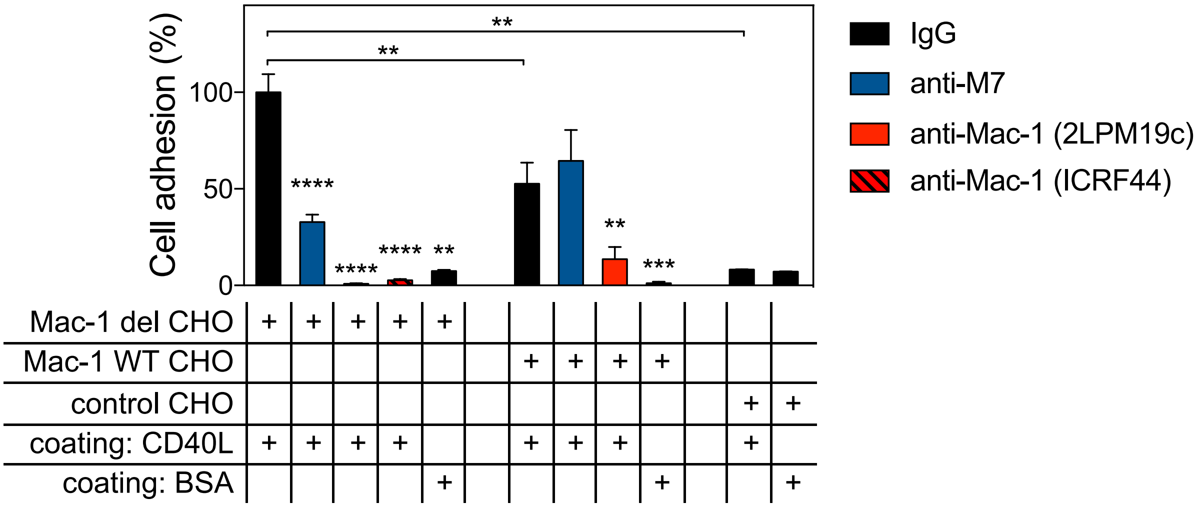

Supplementary Figure 2: Anti-M7 specifically blocks the interaction of the activated, open-conformation integrin Mac-1 with immobilized CD40L. Human sCD40L (CD40L) was coated on 96-well plates (10μg/ml), blocked with 1%BSA/PBS, and incubated with CHO-cells expressing constitutively activated, human Mac-1 (Mac-1 del CHO)[1], CHO cells expressing the human wildtype integrin (Mac-1 WT CHO), or naïve CHO cells that do not express Mac-1 (control CHO) were used. As a control for CD40L, plates were coated with 1% BSA/PBS (BSA). Cells were pre-incubated with blocking antibodies (10μg/ml) against the CD40L-binding site (anti-M7) or against the entire Mac-1 ligand binding I-domain (anti-human Mac-1). Two blocking anti-human Mac-1 antibody clones were tested: 2LPM19c and ICRF44. These clones do not cross-react with mouse Mac-1. Cells were allowed to adhere for 50 minutes. Adhering cells were counted after repeated washing with PBS and were normalized to % of IgG control (Mac-1 del CHO adhesion on CD40L). Error bars indicate mean ± SEM. Statistical significance was tested for the following groups by an unpaired, two-sided Student's T-test: IgG antibody treatment against other antibody treatments of the same CHO-cell type. In addition, significance was tested between control IgG-treated naïve CHO cells and control IgG-treated Mac-1 del CHO or control IgG-treated Mac-1 WT CHO (indicated by brackets), * P < 0.05, ** P < 0.01, *** P < 0.001, **** P <0.0001. N≥4 per group of three individual experiments.

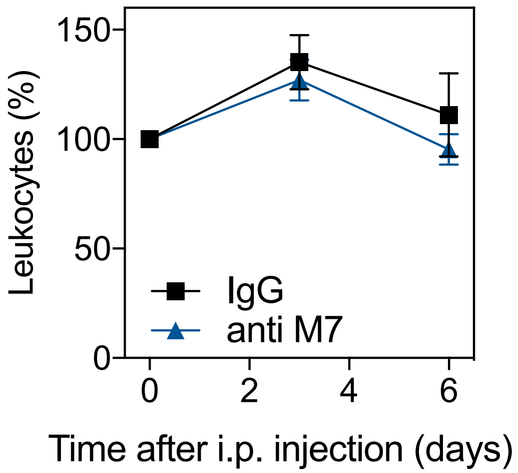

Supplementary Figure 3: Anti-M7 does not reduce peripheral leukocyte counts after i.p. injection. Anti-M7 was injected i.p. in male, C57Bl/6J mice and peripheral leukocyte counts were quantified before (0 days) and 3 and 6 days after injection. Leukocyte counts were normalized to counts before injections in each individual animal (expressed as %). Error bars indicate mean ± SEM. Statistical significance was tested by an unpaired, two-sided Student's T-test between IgG and anti-M7 injected mice at each time point. N=5 animals were included per group.

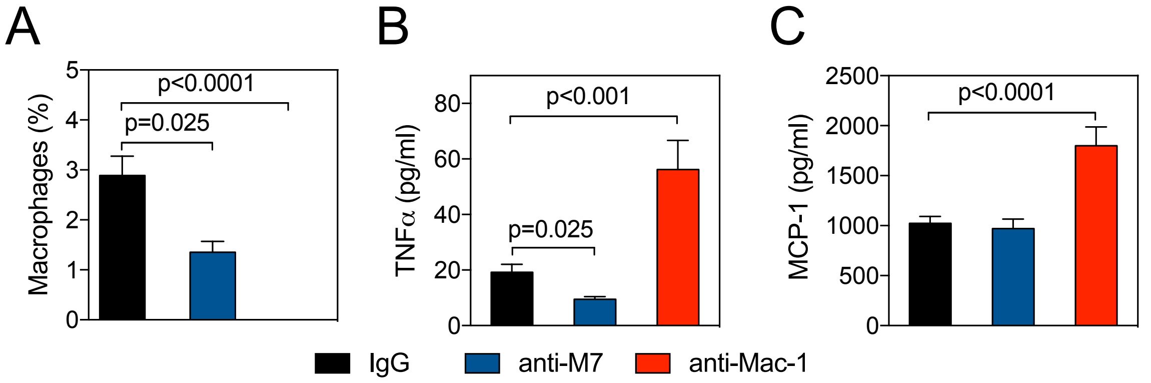

Supplementary Figure 4: Anti-M7 prevents the accumulation of macrophages and dampens inflammatory gene expression in the peritoneal cavity during sterile peritonitis. 8-week-old, male C57Bl/6J mice were injected with 2ml 4% thioglycollate broth i.p. to induce a sterile peritonitis with an accumulation of inflammatory cells in the peritoneal cavity. Simultaneously, 50 μg of F_{ab}-fragment preparations of the following antibodies were injected i.p.: Unspecific IgG isotype-control, anti-M7, or conventional anti-mouse Mac-1 (anti-mouse clone M1/70). After 72 hours, peritoneal cells were collected with a peritoneal lavage and characterized by flow cytometry. Macrophages were identified as $F4/80^{+}Ly\text{-}6G^{-}$ viable $CD45^{+}$ leukocytes. Macrophage content was expressed as percentage of all viable leukocytes (**A**). Concentrations of the cytokines TNFα (**B**) and MCP-1 (**C**) were quantified by a cytometric bead array (CBA). Error bars indicate mean ± SEM. Significance was assessed by an unpaired, two-sided Student's T-test between the indicated conditions. N≥6 mice per group.

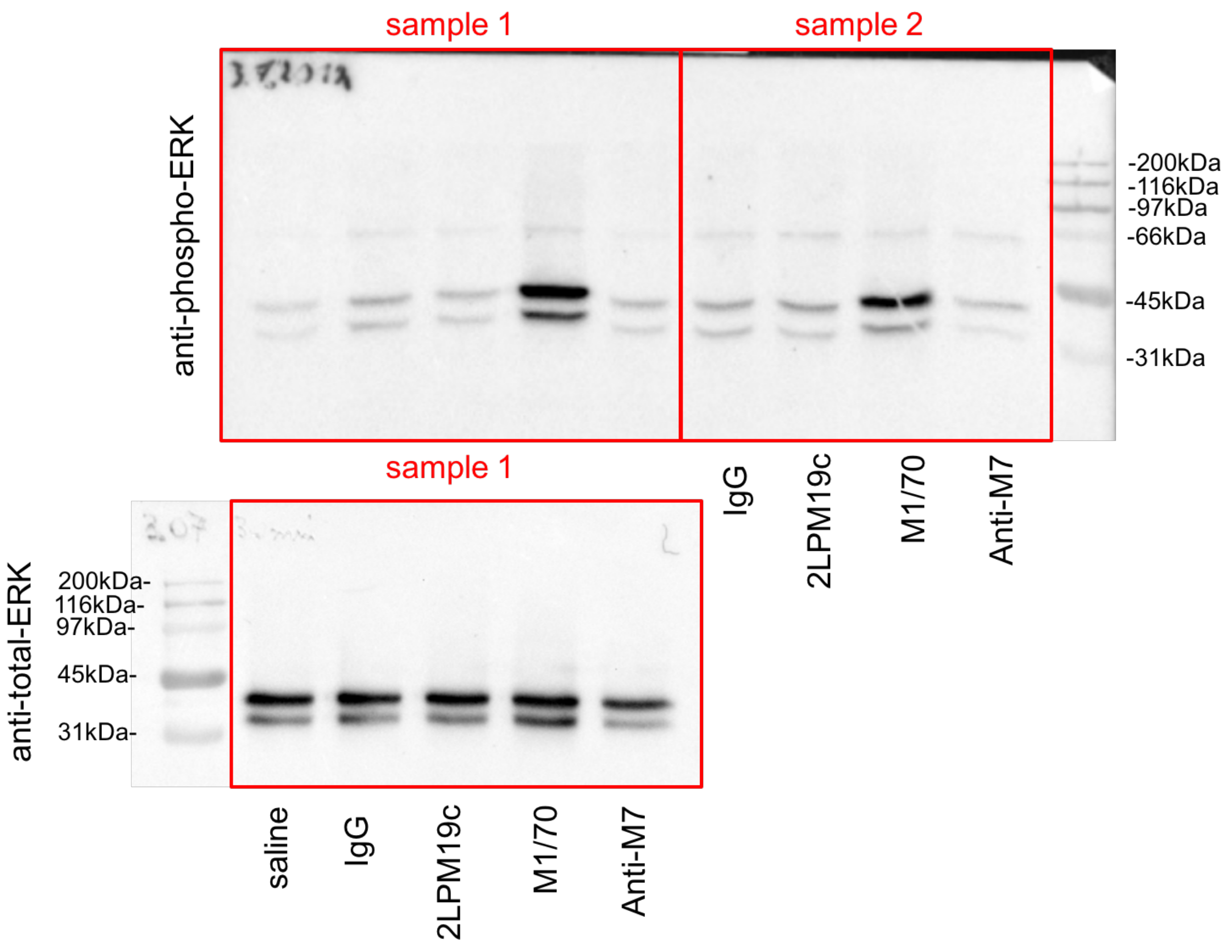

Supplementary Figure 5: Uncropped western blot membranes of Fig. 3A: ERK. Mouse macrophages were isolated from the peritoneal cavity of C57Bl/6 mice 72 hours after injection of 4% thioglycollate. Peritoneal cells were collected by lavage and the purity was confirmed by flow cytometry (>90% F4/80$^+$ macrophages). Cells were cultured in 5%FCS/RPMI overnight and co-incubated with 10μg/ml of mouse IgG, anti-human Mac-1 (clone 2LPM19c), anti-mouse Mac-1 (clone M1/70), or anti-M7 for 30min in the presence of an anti-mouse CD16/CD32 F_c-block. Cells were lysed and total and phosphorylated ERK1/2 was visualized by Western blot.

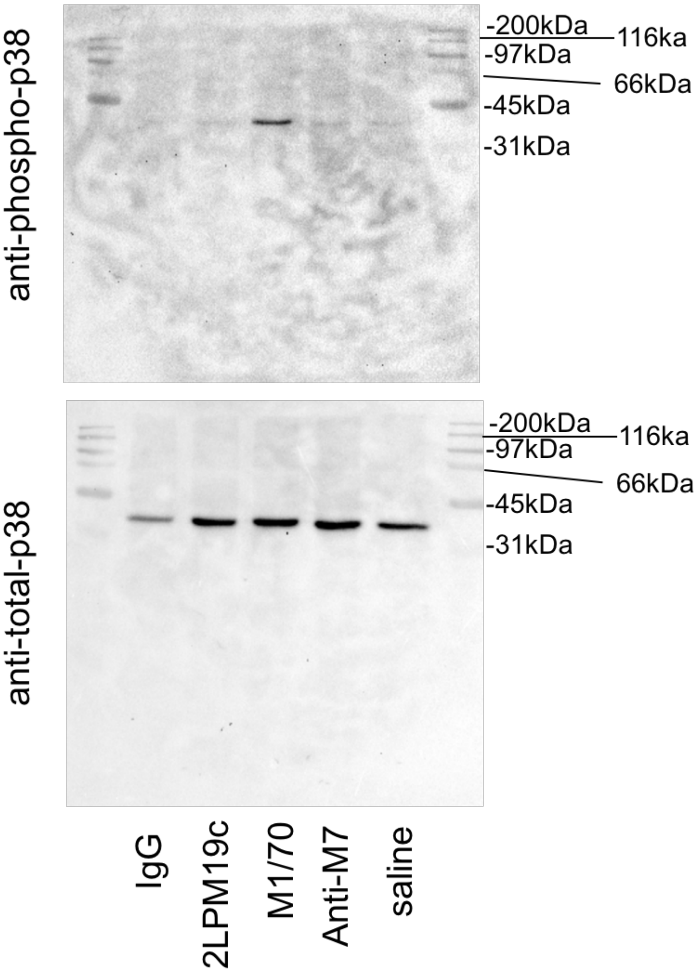

Supplementary Figure 6: Uncut western blot membranes of Fig. 3A: p38. Mouse macrophages were isolated from the peritoneal cavity of C57Bl/6 mice 72 hours after injection of 4% thioglycollate. Peritoneal cells were collected by lavage and the purity was confirmed by flow cytometry (>90% F4/80$^+$ macrophages). Cells were cultured in 5%FCS/RPMI overnight and co-incubated with 10μg/ml of mouse IgG, anti-human Mac-1 (clone 2LPM19c), anti-mouse Mac-1 (clone M1/70), or anti-M7 for 30min in the presence of an anti-mouse CD16/CD32 F_c-block. Cells were lysed and total and phosphorylated p38 was visualized by Western blot.

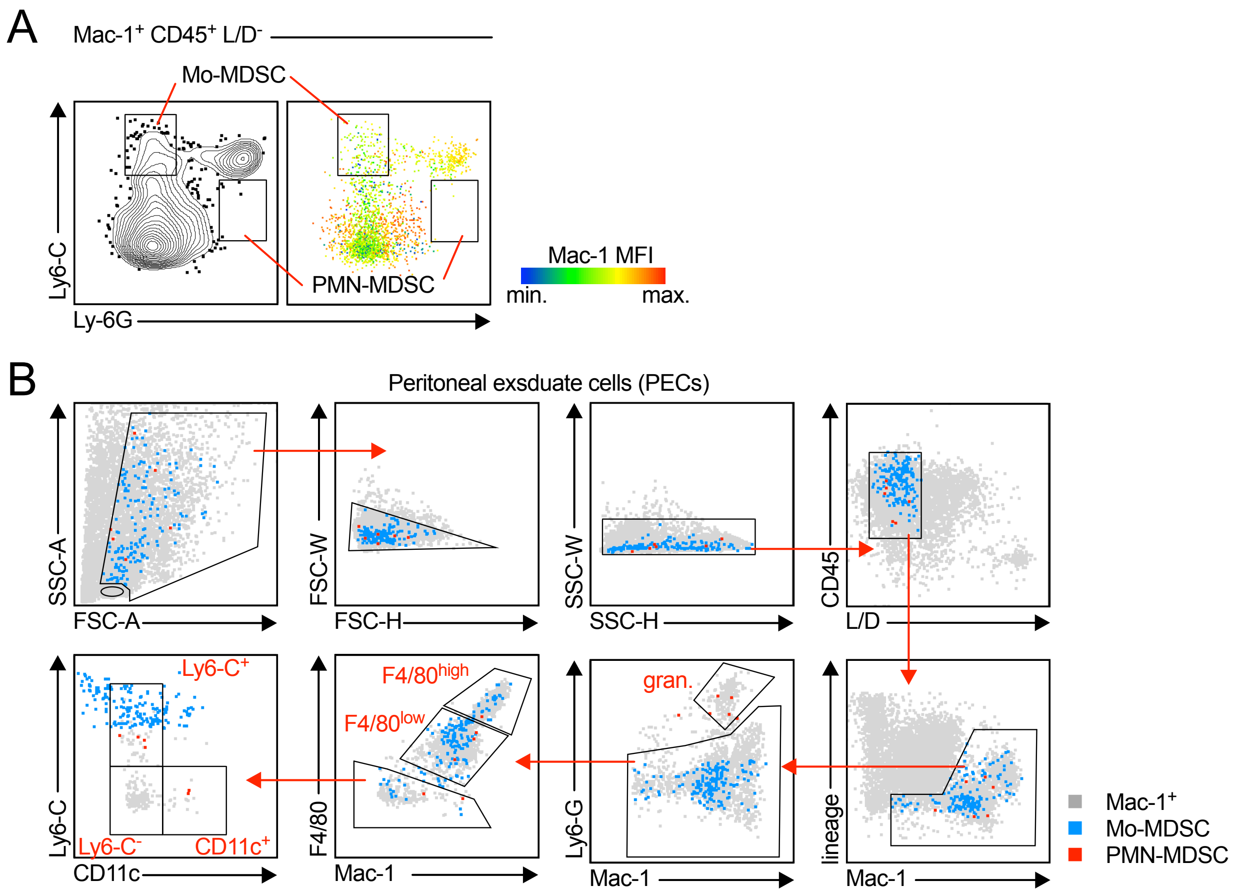

Supplementary Figure 7: Identification of Myeloid-derived Suppressor Cells (MDSCs) in the peritoneal cavity by flow cytometry. Peritoneal cells were collected from 8-week-old, male C57Bl/6J mice by a peritoneal lavage 20 hours after an i.p. injection of 20µg LPS (0111:B4, Invivogen ultra-pure) and 50µg of the indicated antibodies (IgG or anti-M7). The two previously described MDSC-subpopulations, Monocyte-precursor (Mo-MDSC) and PMN-precursor (PMN-MDSC), were gated within viable, CD45$^+$, Mac-1$^+$ leukocytes based on Ly6-G and Ly6-C expression (**A**, left graph). Relative expression of Mac-1 (within Mac-1$^+$ leukocytes with a color code from Mac-1$^{+\,min.}$ to Mac-1$^{+\,max.}$ is shown in (**A**, right graph). Color-coded backgating of both MDSC-subsets within the gates applied to define and quantify myeloid cell populations in the peritoneal cavity (Main Fig. 4). Representative plots are shown in (**A, B**).

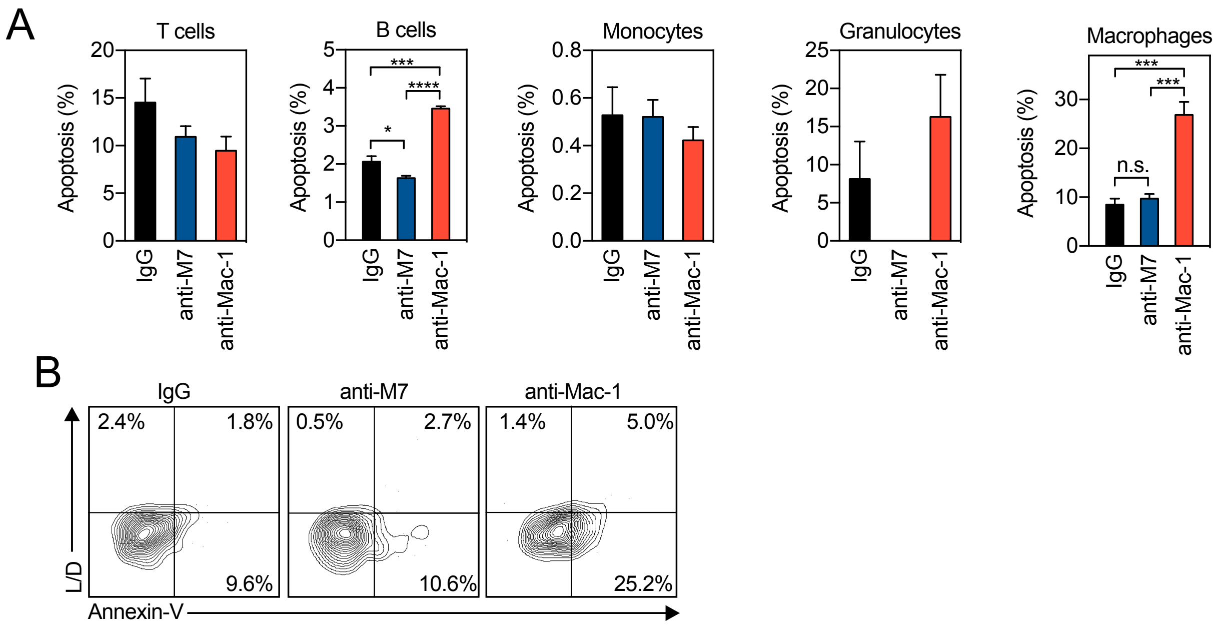

Supplementary Figure 8: Treatment with an unspecific anti-Mac-1 antibody, but not with anti-M7, induces apoptosis and cell death in peritoneal macrophages and B cells. Peritoneal cells were collected from untreated, male 8-week-old C57Bl/6J mice by a peritoneal lavage. 400.000 leukocytes were transferred into a 96-well plate in 50 μl complete RPMI cell culture media containing 1:125 cell stimulation cocktail with PMA and Ionomycin (eBioscience) per well. The indicated antibodies (anti-mouse clone M1/70 as anti-Mac-1) were added to the wells at a final concentration of 20μg/ml. After 4 hours, cells were stained for flow cytometry with an antibody cocktail containing Annexin-V-FITC and a live/dead dye (L/D). Apoptotic cells were identified as L/D$^-$Annexin-V$^+$ and quantified as % of all cells (**A**). Representative FACS plots of macrophage Annexin-V binding are shown in (**B**). Error bars indicate mean ± SEM. Significance was assessed by an unpaired, two-sided Student's T-test. * P < 0.05, ** P < 0.01, *** P < 0.001, **** P <0.0001. N=5 donor mice per group. n.s.: not significant.

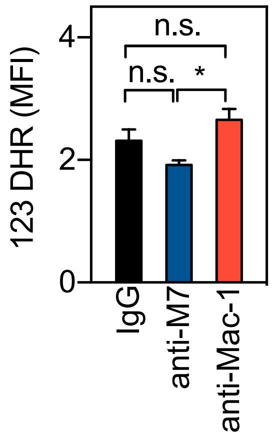

Supplementary Figure 9: Anti-Mac-1 antibodies do not alter the production of reactive oxygen species (ROS) in macrophages. Peritoneal cells were collected from untreated, male 8-week-old C57Bl/6J mice by a peritoneal lavage. 400.000 cells were transferred into a 96-well plate in 50μl complete RPMI cell culture media containing 2μg/ml LPS (0111:B4, Invivogen ultra-pure) per well. The indicated antibodies were added to the wells at a final concentration of 20μg/ml (anti-mouse clone M1/70 as anti-Mac-1). After 4 hours, 123-Dihydro-Rhodamine (123 DHR) was added to the cells and incubated for 30 minutes at 37°C. Cells were washed and stained for flow cytometry. Macrophages were identified as $F4/80^{+}CD11b^{+}Gr\text{-}1^{-}CD45^{+}$ cells. The mean fluorescence (MFI) for 123 DHR was quantified. Error bars indicate mean ± SEM. Significance was assessed by an unpaired, two-sided Student's T-test. * $P < 0.05$. N=5 donor mice per group. n.s.: not significant.

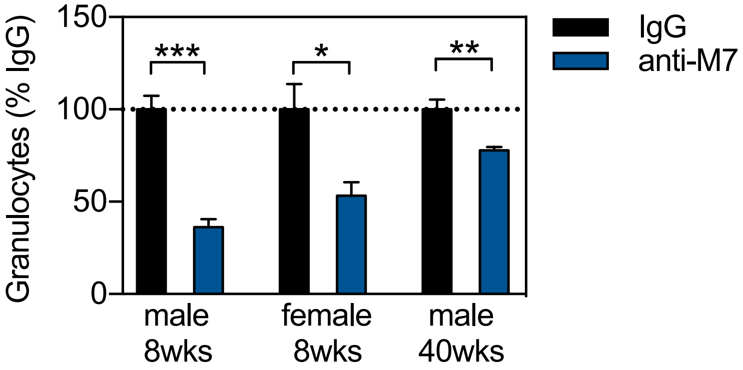

Supplementary Figure 10: Anti-M7 prevents the accumulation of granulocytes in the peritoneal cavity independent of gender and age. Cecal-ligation and puncture (CLP) sepsis was induced in 8-week-old male or female or 40-week-old male C57Bl/6J mice. 50μg of the indicated F_{ab}-antibody preparations (IgG or anti-M7) were injected i.p. 1 hour before CLP surgery. 20 hours after CLP surgery, the number of granulocytes ($Ly6\text{-}G^{+}Mac\text{-}1^{+}F4/80^{-}$) in the peritoneal cavity was quantified and expressed as % of IgG control in the same treatment group. Error bars indicate mean ± SEM. Significance was assessed by an unpaired, two-sided Student's T-test. * $P < 0.05$, ** $P < 0.01$, *** $P < 0.001$. N≥5 mice per group.

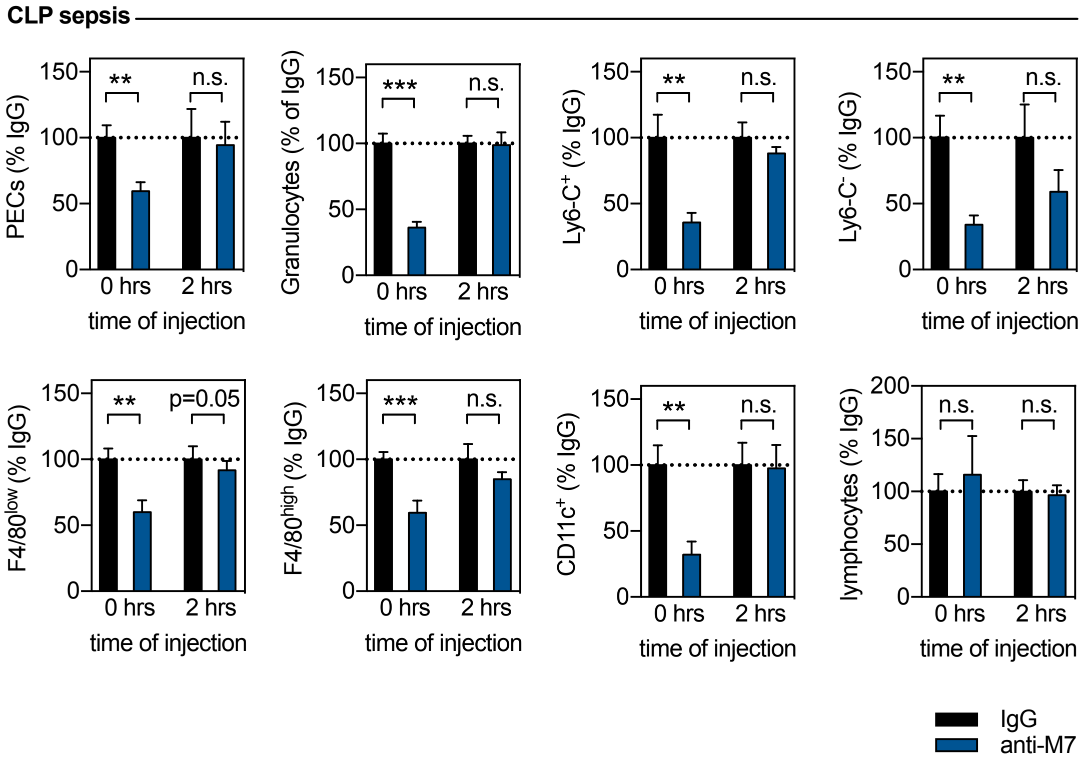

Supplementary Figure 11: Only immediate, but not delayed, treatment with anti-M7 protects from peritoneal leukocyte accumulation in CLP-induced abdominal sepsis. Cecal-ligation and puncture (CLP) sepsis was induced in 8-week-old, male C57Bl/6J mice. 50μg of the indicated F_{ab}-antibody preparations (IgG or anti-M7) were injected i.p. 1 hour before surgery (0 hours) or with a delay of 2 hours. 20 hours later, the number of peritoneal exudate cells (PECs), granulocytes (Ly6-G^+ Mac-1^+ F4/80^-), inflammatory (Ly6-C^+) and patrolling (Ly6-C^-) monocytes, F4/80^{low} and F4/80^{high} macrophages (Mac-1^+ Ly-$6G^-$), myeloid derived dendritic cells ($CD11c^+$), and lymphocytes was quantified and expressed as % of IgG control in the same treatment group. Error bars indicate mean ± SEM. Significance was assessed by an unpaired, two-sided Student's T-test. ** $P < 0.01$, *** $P < 0.001$. N≥5 mice per group. n.s.: not significant.

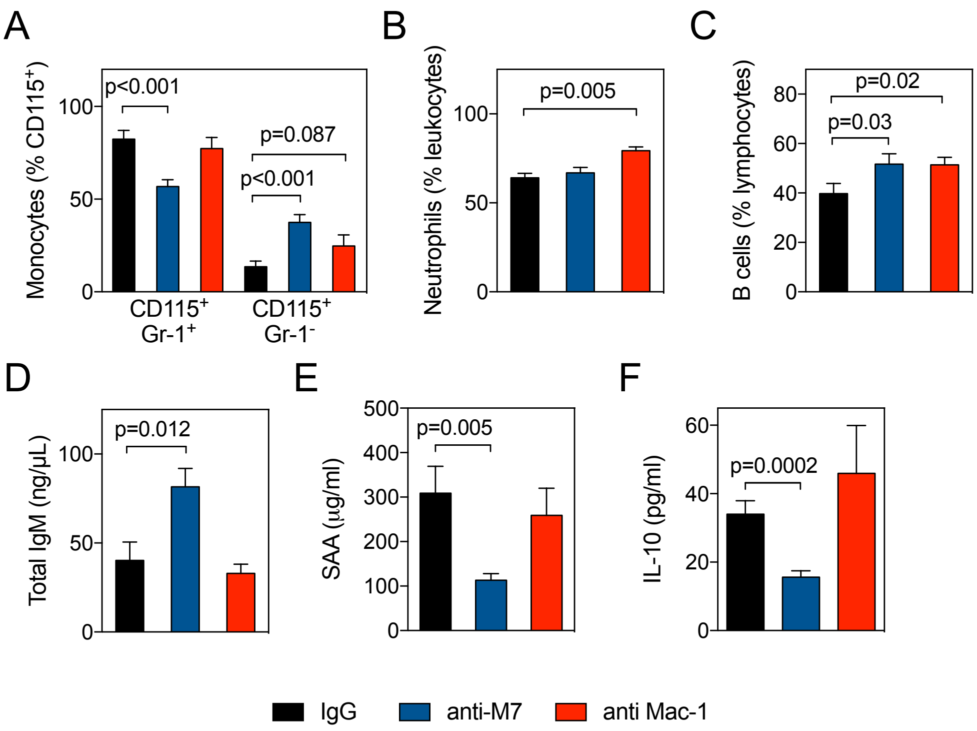

Supplementary Figure 12: The CD40L/Mac-1 interaction improves innate immunity during polymicrobial sepsis. Cecal-ligation and puncture (CLP) sepsis was induced in male, 8-week-old C57Bl/6J mice. 50µg of the indicated F_{ab}-antibody preparations were injected i.p. 1 hours before CLP surgery. 20 hours after CLP surgery, peripheral leukocytes were quantified in blood samples by flow cytometry: Inflammatory (Ly6-C$^+$) and patrolling (Ly6-C$^-$) CD115$^+$ Mac-1$^+$ monocytes (**A**), Ly6-G$^+$ CD115$^-$ Mac-1$^+$ neutrophils (**B**), and CD19$^+$ B cells (**C**). Cell numbers were expressed as % of all CD45$^+$ leukocytes. (**D**) Levels of circulating IgM antibodies. (**E**) Plasma levels of the acute-phase protein Serum-Amyloid A (SAA) and of (**F**) IL-10 were quantified by ELISA in plasma samples. Error bars indicate mean ± SEM. Significance was assessed by an unpaired, two-sided Student's T-test. N≥10 mice per group.

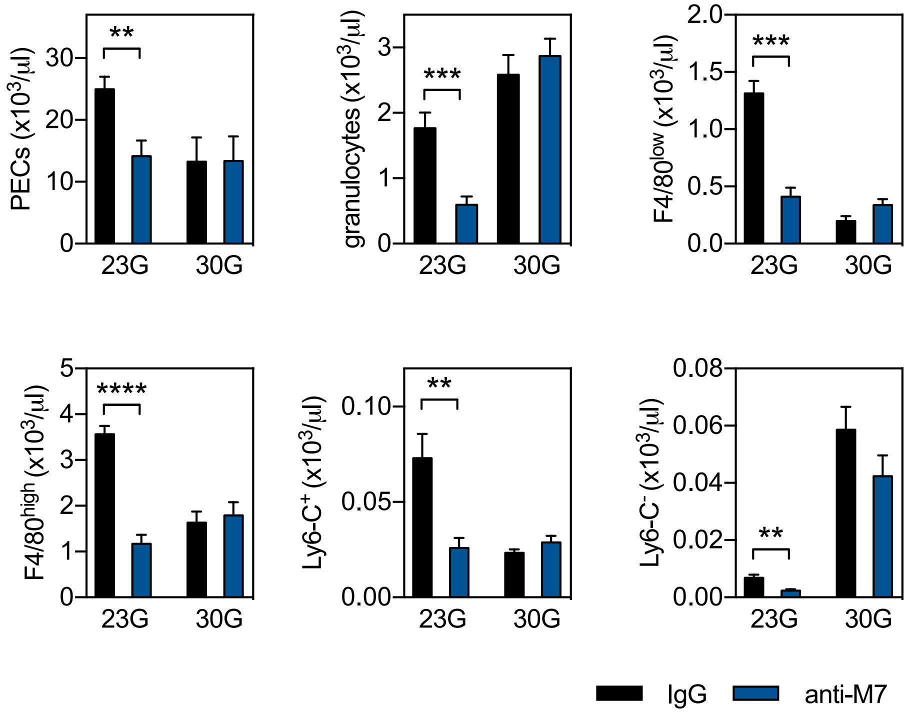

Supplementary Figure 13: Anti-M7 does not modulate low-grade inflammatory cell accumulation in moderate CLP-30G sepsis. Cecal-ligation and puncture (CLP) sepsis was induced in 8-week-old male C57Bl/6J mice. For the puncture, a standard 23G needle or a 30G needle with a smaller outlet was used. 50μg of F_{ab}-preparations of the indicated antibodies (IgG or anti-M7) were injected i.p. one hour before surgery. 20 hours after CLP surgery, the number of peritoneal exudate cells (PECs), granulocytes (Ly6-G^+ Mac-1^+ F4/80^-), inflammatory (Ly6-C^+) and patrolling (Ly6-C^-) monocytes, F4/80^{low} and F4/80^{high} macrophages (Mac-1^+ Ly-$6G^-$), and myeloid derived dendritic cells ($CD11c^+$) in the peritoneal cavity was quantified. Error bars indicate mean ± SEM. Significance was assessed by an unpaired, two-sided Student's T-test. ** P < 0.01, *** P < 0.001, **** P < 0.0001. N≥5 mice per group.

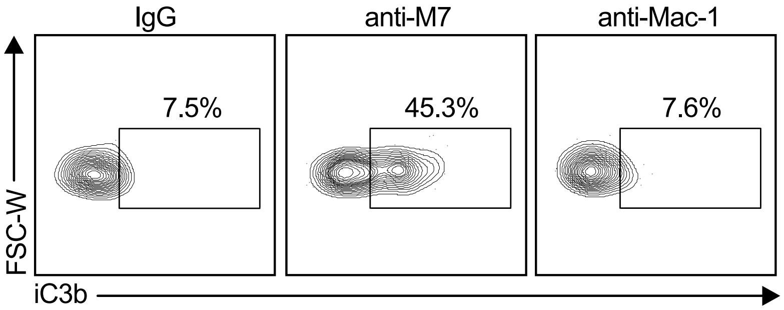

Supplementary Figure 14: Binding of the complement factor iC3b to peritoneal macrophages is enhanced after an *in vitro* treatment with anti-M7. Peritoneal cells were collected from untreated, male 8-week-old C57Bl/6J mice by a peritoneal lavage. 400.000 leukocytes were transferred into a 96-well plate in 50 µl complete RPMI cell culture media containing 1:125 cell stimulation cocktail. Fresh rat serum (containing iC3b) in a final concentration of 10% was added. The indicated antibodies (anti-mouse clone M1/70 as anti-Mac-1) were added to the wells at a final concentration of 20µg/ml. After 4 hours incubation at 37°C, cells were stained for flow cytometry with an antibody cocktail containing anti-iC3b. Macrophages were identified as F4/80^{+}CD11b^{+}Gr-1^{-}CD45^{+} cells. A representative flow cytometry contour plot of anti-iC3b gated on peritoneal macrophages is shown.

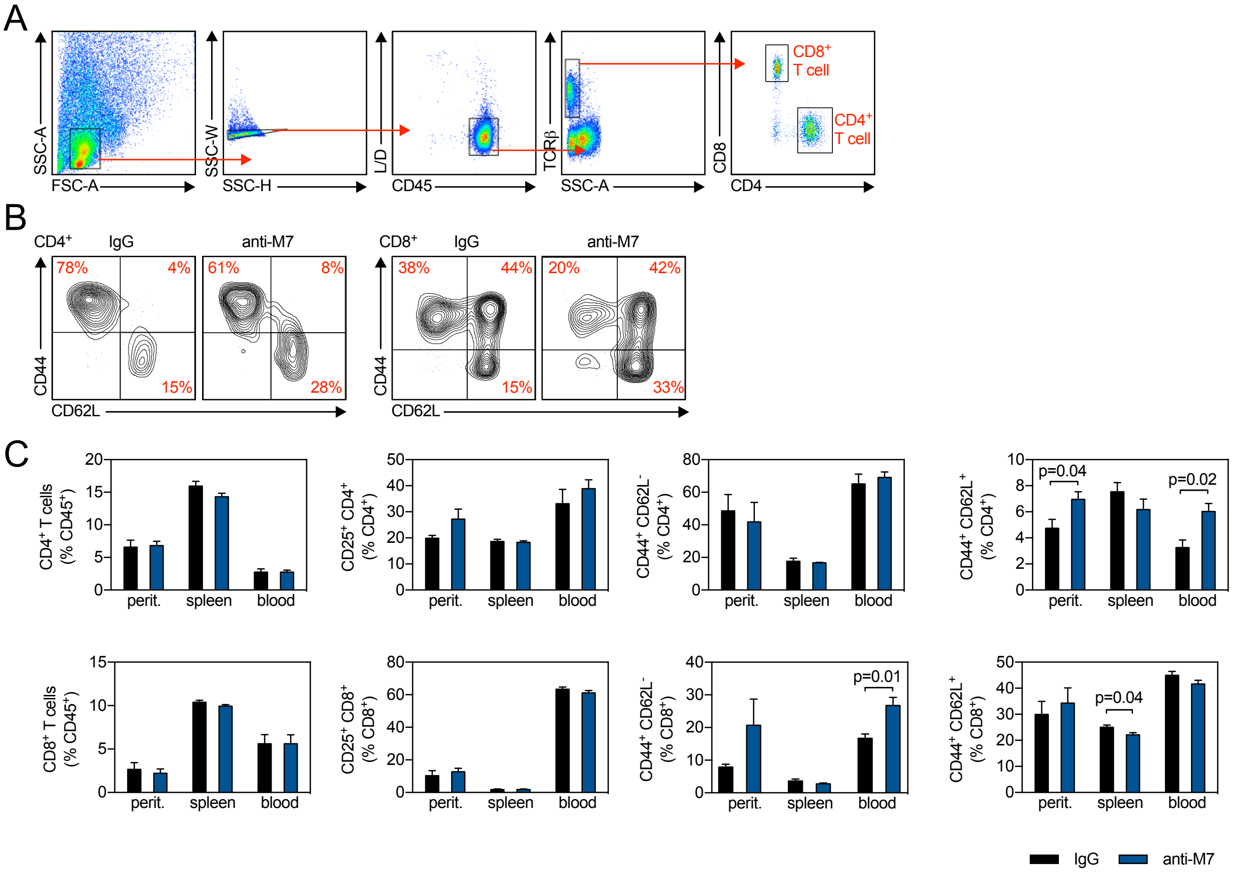

Supplementary Figure 15: Anti-M7 induces a state of enhanced T cell activation. Peritoneal cells (perit.), splenocytes (spleen), or blood leukocytes were collected from male, 8-week-old C57Bl/6J mice 20 hours after an i.p. injection of 20µg LPS (0111:B4) and 50µg of the indicated antibodies (IgG or anti-M7). Cells were stained for flow cytometry and T cells were identified as $CD45^{+}L/D^{-}TCR\text{-}\beta^{+}CD4/8^{+}$ single cells in the lymphocyte gate (**A**). The markers L-Selectin (CD62L) and CD44 were used to determine the activation state of T cells: T-effector memory cells (T_{EM}, $CD44^{+}CD62L^{-}$), T-central memory cells (T_{CM}, $CD44^{+}CD62L^{+}$), naïve T cells ($T_{naïve}$, $CD44^{-}CD62L^{-}$). In addition, CD25 was used as an activation marker (**B**). Percentage of T_{EM}, T_{CM}, T_{naive}, and $CD25^{+}$ T cells of all $CD4^{+}$ or $CD8^{+}$ T cells from the different sources. Error bars indicate mean ± SEM. Significance was assessed by an unpaired, two-sided Student's T-test between IgG and anti-M7 treated groups in the same location. N≥5 donor mice per group.

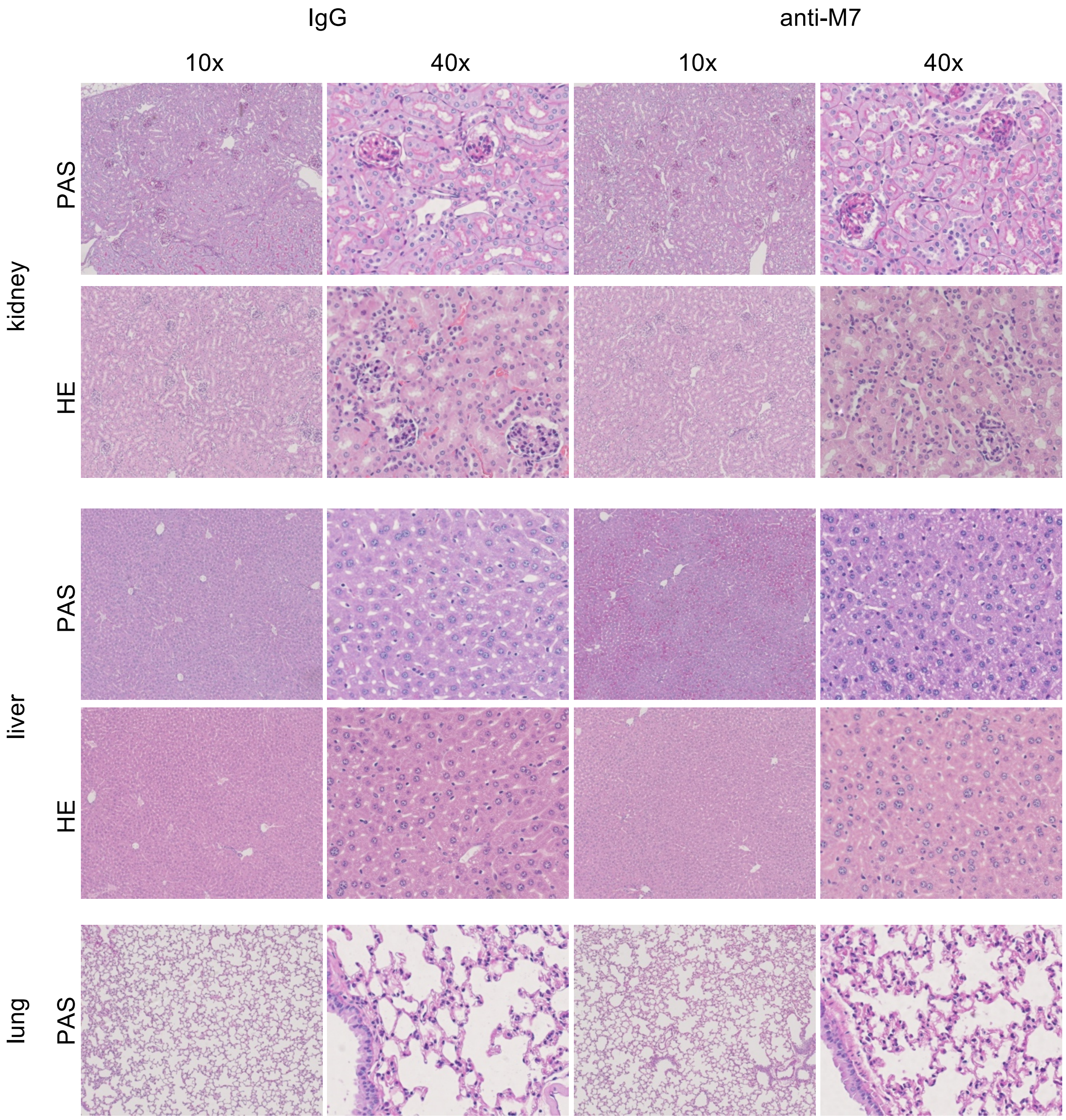

Supplementary Figure 16: anti-M7 does not impact on sepsis-related organ damage 20 hours after initiation of abdominal sepsis. Cecal-ligation and puncture (CLP) sepsis was induced in male, 8-week-old C57Bl/6J mice. 50μg of F_{ab}-preparations of the indicated antibodies were injected i.p. one hour before surgery. 20 hours after CLP surgery, organs were excised, fixed in 4% PFA, sliced, and stained with HE or PAS. Representative tissue sections from 10 mice per group are shown.

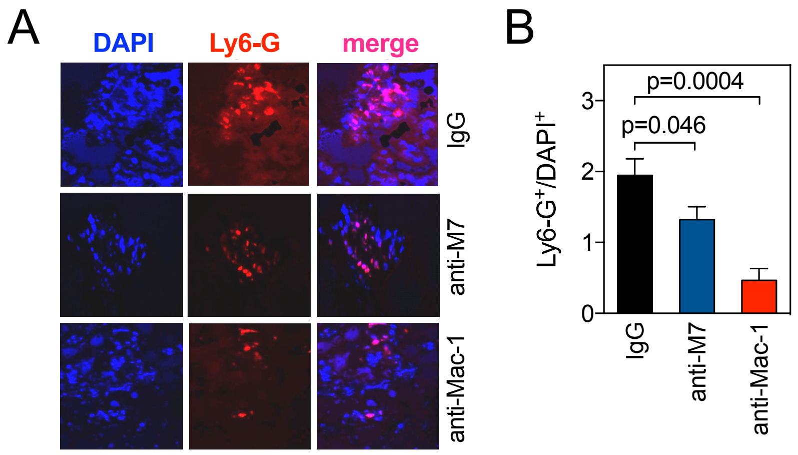

Supplementary Figure 17: Anti-M7 reduces the accumulation of granulocytes in the kidney during abdominal sepsis. Cecal-ligation and puncture (CLP) sepsis was induced in male, 8-week-old C57Bl/6J mice. 50μg of F_{ab}-preparation of the indicated antibodies (IgG, anti-M7, or the anti-mouse anti-Mac-1 clone M1/70) were injected i.p. one hour before CLP surgery. 20 hours after CLP surgery, kidneys were excised, fixed in 4% PFA, sliced, and stained with DAPI, and an antibody against the granulocyte epitope Ly6-G. In fluorescent microscopy, granulocytes were identified as $DAPI^+Ly6\text{-}G^+$ cells (**A**). Error bars indicate mean ± SEM (**B**). Significance was assessed by an unpaired, two-sided Student's T-test between the indicated groups. N≥10 mice per group.

SUPPLEMENTARY REFERENCES

1. Schuler P, Assefa D, Ylanne J, Basler N, Olschewski M, Ahrens I, Nordt T, Bode C, Peter K. Adhesion of monocytes to medical steel as used for vascular stents is mediated by the integrin receptor mac-1 (cd11b/cd18; alpham beta2) and can be inhibited by semiconductor coating. *Cell Commun Adhes*. 2003;10:17-26
